# The Dynamic Impact of Natural Resource Rents, Financial Development, and Technological Innovations on Environmental Quality: Empirical Evidence from BRI Economies

**DOI:** 10.3390/ijerph19010130

**Published:** 2021-12-23

**Authors:** Siming Zuo, Mingxia Zhu, Zhexiao Xu, Judit Oláh, Zoltan Lakner

**Affiliations:** 1 School of Economics, Beijing Technology and Business University, Beijing 100029, China; simingzuo@163.com; 2 School of International Trade and Economics, University of International Business and Economics, Beijing 100029, China; 3 Graduate School, University of International Business and Economics, Beijing 100029, China; xuzhexiao@uibe.edu.cn; 4 Faculty of Economics and Business, University of Debrecen, 4032 Debrecen, Hungary; olah.judit@econ.unideb.hu; 5 Department of Public Management and Governance, College of Business and Economics, University of Johannesburg, Johannesburg 2006, South Africa; 6 Department of Food Economics, Faculty of Food Science, Hungarian University of Agriculture and Life Science (MATE), 2100 Godollo, Hungary; Lakner.Zoltan.Karoly@uni-mate.hu

**Keywords:** natural resources rents, technological innovation, financial development, Environmental Kuznets Curve (EKC), ecological footprint, Belt and Road Initiative

## Abstract

Until recently, many countries’ policies were motivated by economic growth; however, few strategies were developed to prevent environmental deterioration including reducing the ecological footprint. In this context, the purpose of this study was to analyze the role of natural resource rents, technological innovation, and financial development on the ecological footprint in 90 Belt and Road Initiative (BRI) economies. This research divided the BRI economies into high income, middle-income, and low-income levels to capture income differences. This research used the second-generation panel unit root, cointegration, and augmented mean group estimators to calculate the robust and reliable outcomes. Based on the annual data from 1991 to 2018, the findings show that natural resource rents drastically damage the quality of the environment, whereas technological innovations are helpful in reducing ecological footprint. Moreover, the outcome of the interaction term (natural resource rents and technological innovations) negatively impacts the ecological footprint. Interestingly, these findings were similar in the three income groups. In addition, financial development improved environmental quality in the middle-income BRI economies, but reduced it in high-income, low-income, and full sample countries. Furthermore, the Environmental Kuznets Curve (EKC) concept has been validated across all BRI economies. Policymakers in BRI countries should move resources away from resource-rich sectors of industries/manufacturing sectors to enhance/promote economic growth and use these NRRs efficiently for a progressive, sustainable environment. Based on these findings, several efficient policy suggestions are proposed.

## 1. Introduction

In recent years, environmental conditions such as pollution, substandard sanitation, and significant loss of natural resource rents (NRRs) and forest reserves have been key concerns for many countries. Meager environmental conditions jeopardize human health and economic well-being. These elements are vulnerable to climate change including health, natural and physical capital, and access to water, food, and land [1]. These environmental problems have sparked a worldwide campaign to resist climate change. However, in recent years, most of the Belt and Road Initiative (BRI) economies have been straining efforts to upgrade their industrial movement, massive combustion of fossil fuel energy in the manufacturing sector, which consequently increases global warming [2]. Researchers have traditionally used carbon (CO_2_) emissions to proxy environmental quality in the current environmental sustainability literature. However, this indicator has been criticized by several scholars as CO_2_ emissions are accountable for a minor portion of the whole environment and do not fully encapsulate environmental pollution. Nathaniel and Khan [3] claim that CO_2_ emissions do not anticipate the stocks of resources (e.g., oil, soil, forest, gas, and petroleum). Therefore, it is necessary to use an inclusive proxy for environmental sustainability that removes the limitation links with CO_2_ emissions and offer suitable insights to policymakers/regular authorities related to the environmental quality. For this situation, the ecological footprint (EF) is a widely recognized proxy for environmental quality that can manage and assess NRRs [4]. Hence, recent empirical literature has used EF to measure environmental quality [3,5,6,7].

The NRR has an essential element of the global economy, specifically most of the BRI economies that depend on extricating them for a significant portion of their economic growth [8]. The NRRs comprise forests, gas, oil, minerals, and coal. However, the link between NRR and environmental quality is very intricate and shows contrasting evidence. As an example, Shen et al. [9]; Hussain et al. [10]; Udi et al. [11]; Wang et al. [12] documented that NRRs positively influence environmental quality, whereas Khan et al. [4]; Adedoyin et al. [13]; Li et al. [14]; Balsalobre-Lorente et al. [15] described a negative association between NRRs and the environmental quality. Certainly, the literature on the association between NRRs and an inclusive environmental proxy such as EF and additional inquiries are essential to moving toward a sustainable environment. NRRs are directly associated with the income level of an economy. In the first stage, people utilize more energy (e.g., more NRRs) for development purposes, which will increase the economic growth and ignore the effects on the environment, but in later stages, when the standard of living improves, they then adopt a cleaner environmental strategy, protection of NRRs, and most concern on energy-efficient products, indicating the presence of an Environmental Kuznets Curve (EKC). Hence, NRRs can significantly enhance environmental quality and boost economic growth [16].

Furthermore, the state of the financial system within an economy is also asserted as a major contributor to its environmental well-being. Usually, although likely to boost economic growth, an underdeveloped financial system could be harmful to the quality of the environment. For example, the less-developed financial systems within the BRI countries can be expected to provide finance for pollution-intensive industries whereby the borrowed funds can be invested in environmentally unfriendly production processes. Consequently, the prospect of achieving environmental sustainability can be largely compromised. Conversely, a developed financial system could be beneficial for extending green finance, which can provide credit to firms keen to invest in energy-efficient production processes [17,18]. Furthermore, the financial services sector can also portray a central part in financing research and development-related projects to achieve technological innovations for environmental development [19]. Hence, it can be said that financial development (FD) is a vital component for ensuring environmental welfare [20].

There are several compelling reasons why we chose to undertake this study in BRI economies. From the start of the BRI in 2013 through to the end of 2019, China invested USD 760 billion, with 39 percent going into the energy industry, roughly 26 percent going into transportation, and 7 percent to metals [21]. In terms of NRRs, the BRI countries have 58.54 percent of proven reserves of crude oil, 53.82 percent of natural gas output, 74.69 percent of total coal output, and 55.17 percent of oil supplies worldwide [10]. Likewise, this initiative reaches 62% of the world’s population. These countries account for 31% of world gross domestic product (GDP), and the share of global trade is 35% [22]. Furthermore, this initiative is accountable for 28% of CO_2_ emissions and a 2 °C increase in global temperature (excluding China). Therefore, assuming development proceeds as projected, CO_2_ emissions will increase by 66% until 2050 [23]. Because of their economic and financial connectivity, the BRI economies have critical economic significance [24]. All these factors combine to make the BRI a viable option for research in environmental economics. The efficient utilization of NRRs is necessary to stimulate economic growth [25]. The green technological innovations can enhance the utilization and allocation of NRRs; they increase the capability of raw materials and the exponential of NRRs to achieve the path of sustainable development [26]. Moreover, the FD helps to enhance the efficiency of NRR extractions using technological innovation (TI) [16]. This justifies the reason for including variables such as NRR, FD, and TI in ecological function, so the objective of this study was to analyze the impact of NRR, FD, TI, and economic growth on EF in BRI economies.

This work contributes to the current literature in the following ways. First, this research examines the effect of NRR, FD, and TI on the EF from 1991–2018 for 90 BRI countries. Furthermore, this study divided the BRI countries into three income levels (i.e., high income, middle income, and low income) to examine the influence of these potential indicators on EF to assess potential disparity in the association between NRR extraction and EF due to their income differences. Second, the present study used the moderating effect of NRR with TI in reducing the EF. It would be helpful to examine whether NRR indicated with TI reduces the overall level of EF in the BRI countries. This moderating effect may help to improve NRR efficiency through TI [5]. Third, this research also examines the EKC hypothesis of BRI countries. Fourth, following confirmation of the possible cross-sectional dependence across cross-sections, this study used a comparatively advanced and robust econometric approach (i.e., CIPS unit root test, Westerlund cointegration approach, and augmented mean group for long-run elasticity), which enhances the efficiency and consistency of our finding. Fourth, although several empirical studies have investigated the NRR-environment, TI-environment, and FD-environment nexus separately, however, none of these studies have examined these links simultaneously in a single model. Consequently, this study adds to the existing literature, where these relationships are scrutinized under separates titles. Finally, this study used greenhouse gas (GHG) emission, another environmental proxy, and matched the outcomes to ensure robustness.

The rest of the paper is structured as follows. Section 2 discusses the literature review of earlier studies. Section 3 explains the theoretical framework, data, and methodology. Section 4 discusses the findings and their interpretation and robustness checks. Finally, Section 5 reveals the conclusion and policy implications.

## 2. Literature Review

Global warming and climate change as well as increased awareness of these problems have increased the importance of understanding environmental quality and its elements. We divided the literature into four sections to elaborate on the relationships between the study’s variables and EF. The first section describes the nexus between NRR-environment; the second section addresses TI-environment; the third section discusses FD-environment; and the fourth section elaborates the relationship between economic growth and environment.

### 2.1. Nexus between Natural Resource Rents (NRR) and Environment

Recently, environmental sustainability and NRRs have received more attention among policymakers and researchers. For example, Ahmad et al. [5] analyzed the relationship between NRR, TI, GDP growth, and environmental quality in twenty-two emerging countries from 1984–2016. The outcome suggested that NRR and GDP growth increased EF, while TI had a favorable influence on environmental quality. Similarly, Erdoğan et al. [27] inspected the dynamic association between NRR, globalization, human capital, urbanization, and EF in twenty-three Sub-Saharan African countries covering 1980–2016. The results revealed that NRR and urbanization enhanced EF, while globalization and human capital improved environmental quality. Likewise, Danish et al. [6] examined the relationship between renewable energy use, NRR, urbanization, and environmental quality in BRICS economies from 1992–2016. Their findings showed that renewable energy, urbanization, and NRRs enhanced environmental quality. 

Balsalobre-Lorente et al. [15] analyzed the association between NRR, GDP growth, and renewable electricity based on five European Union countries from 1985–2016. The results of the study specified that renewable electricity and NRR improved environmental sustainability. However, Khan et al. [28] investigated the NRR, tourism, energy use, and environmental quality nexus in 51 BRI economies from 1990–2016. The study’s findings revealed that NRR is causally linked to tourism, energy use, and environmental quality in these economies. Between the years 1970–2016, Ahmed et al. [29] revealed that NRR and urbanization intensified the quality of the environment. In contrast, human capital has a positive effect on the environmental quality in the case of China. However, Zafar et al. [30] showed the negative relationship between NRR and environmental quality due to eco-friendly technologies. To sum up, after discussing the literature review and focusing on the influence of NRR on environmental sustainability, the effect of NRRs on the environment varies from country and time disparities. Given the empirical/theoretical settings, we present the following first testable hypothesis.

**H1.**  *NRR has an ambiguous effect on the EF in BRI economies.*

### 2.2. Nexus between Technological Innovation (TI) and Environment

According to endogenous growth theory, research and development (R&D) expenditures can boost economic productivity and NRR utilization, however, the involvement of TI in environmental sustainability, especially EF, is uncertain [31]. Chen and Lee [32] used the ordinary least squares (OLS) and fixed effect method to look into the connection between globalization, TI, and the environment in ninety-six economies from 1970–2016. The findings demonstrate that TI has a favorable effect in diminishing environmental damage. Kumail et al. [33] examined the dynamic relations between TI and environmental sustainability in Pakistan from 1990–2017. The findings show that TI enhanced the environmental quality. 

Likewise, Ke et al. [34] studied the causal link between the TI and EF for 280 Chinese cities from 2014–2018. The outcomes of this study revealed that TI increases the environmental quality. Ganda [35] examined TI and environmental quality and found that TI enhanced the environmental quality through investment in the R&D sector. Most researchers believe that TI is favorable to maximizing environmental quality [12,36,37,38]. They argue that TI introduces the efficient progression of new technological applications. Therefore, it directly enhances energy efficiency and reduces the demand for fossil fuel energy utilization. Therefore, it improves the environmental quality. Alternatively, other researchers believe that TI might positively impact environmental quality [35,39,40,41]. In summary, the impact of TI on environmental quality is controversial, could be positive/negative, and the academic literature still has not reached any definite conclusions. Given the empirical/theoretical settings, we present the second hypothesis as follows:

**H2.**  *TI has a negative effect on EF in BRI economies.*

### 2.3. Nexus between Financial Development (FD) and Environment

The existing literature has documented mixed environmental impacts associated with FD. Among the studies that have claimed FD to be detrimental to environmental well-being, Yasin et al. [42] concluded that FD, although increasing the EF levels in the developed countries, is able to reduce the long-run EF figures of the less-developed nations. Hence, the authors advocated that the growth of the financial sector is a relatively more credible means of achieving environmental sustainability in developing countries than in developed ones. Conversely, Mrabet and Alsamara [43] concluded that FD degrades environmental quality in Qatar by boosting the EF figures in both the short and long run. In another similar study on 11 newly industrialized economies, Destek and Sarkodie [44] used the augmented mean group (AMG) method to scrutinize the FD–EF nexus. The findings reported were heterogeneous as FD and EF, in the long run, were ascertained to be positively correlated for the case of Singapore, negatively correlated for the cases of China and Malaysia, and statistically insignificant for the entire panel, Brazil, India, Mexico, Philippines, South Africa, South Korea, Thailand, and Turkey. On the other hand, Naqvi et al. [45] recently found evidence of FD being ineffective in influencing the EF levels in low, lower-middle, and upper-middle-income nations in the long run. On the basis of the relevant studies on the FD–EF nexus, the following is the third hypothesis that will be tested in this study:

**H3.**  *The effect of FD on EF is ambiguous in BRI economies.*

### 2.4. Nexus between Economic Growth and Environment

Since the pioneering work of Grossman and Krueger [46], investigations regarding the association between economic growth and environmental deterioration have centered on the EKC hypothesis supposing an inverted U-shaped influence of economic growth on environmental quality. Furthermore, Stern [47] suggested that if the level of real income enhances, then it demands an improvement in environmental excellence because people will adopt the latest technologies in a production process to protect the environment. In this pursuit, several researchers have found a U-shaped EKC hypothesis: Danish et al. [6] for the BRICS economies; Ahmad et al. [5] for 22 emerging economies; and others have found an inverted U-shaped EKC hypothesis, for instance, Wang et al. [48] for 150 economies; Altıntaş and Kassouri [49] for 14 EU economies; Destek and Sarkodie [44] for 11 economies; Bello et al. [50] for Malaysia; Khoshnevis Yazdi and Ghorchi Beygi [51] for 25 Africa countries; Ma et al. [52] for France and Germany. Based on the EKC hypothesis literature, the study led to our fourth hypothesis, which can be stated as follows:

**H4.**  *There is an inverted U-shape relationship between economic growth and EF in BRI economies.*

### 2.5. Literature Summary

Therefore, after summarizing the relevant studies in the existing literature, the following gaps in the empirical literature can be identified: (a) it is clear that not many research articles have applied the EF for environmental quality assessment purposes in the context of large samples of BRI economies; (b) the literature concerning panel studies on the TI–EF nexus in the context of BRI economies is limited as the existing studies are mostly country-specific or have used short panels; (c) the previous studies have primarily discovered the individual (isolated) impacts of NRR, and TI on EF, while the joint (interactive) impacts of these variables have not been analyzed much in the previous studies; and (d) it is evident that the existing studies have conventionally used panel data estimators. Against this milieu, this study aims to bridge these above-mentioned gaps in the literature using longitudinal/panel data from 1991 to 2018 of 90 BRI economies.

## 3. Theoretical Framework, Data, and Estimation Techniques

From a theoretical perspective, we looked at the association between NRR, TI, FD, and the EF on the treadmill production theory, endogenous growth theory, and ecological modernization theory. The treadmill theory of the product claims that environmental pollution directly affects NRR and economic growth [53]. Endogenous growth and ecological modernization theories provide the idea that TI and FD have more ability to support economies with sustainable economic growth in favor of environmental quality [5]. According to Spaargaren and Mol [54], ecological modernization theory was constructed on the principle of turning around how modern industrial societies deal with environmental problems. However, the rapid increase in production and economic growth reduces the NRR and successively destroys environmental quality due to weak environmental regulations.

The EKC hypothesis is widely employed in the existing literature to examine environmental sustainability due to economic growth. It is assumed that the three different channels of GDP growth that affect environmental sustainability are scale effect, composition effect, and technique effect [55]. According to the scale effect, GDP has a negative influence on environmental quality where economic activities increase the volume of production level that will decrease environmental quality. Furthermore, the composition effect is based on the industry structure of an economy; for example, at the initial stages, environmental quality with changes in economic reforms such as heavy industries (resource-intensive heavy manufacture industries). Finally, the technique effect recommends that outdated technologies are substituted by eco-friendly and updated capital that increases the environmental performance. Therefore, based on the EKC hypothesis, the negative influences of scale effects on the environmental quality will be dominant at the early stages of the economic growth process. However, the positive consequences of composition and technique effects lead to an improvement in environmental quality [47]. Using the assertions made above, we developed the following model to explain the influence of NRR, TI, and FD on the EF in the case of 90 BRI economies.

(1) $$\mathrm{EF}=f\;\left(\mathrm{NRR},\;\mathrm{TI},\;\mathrm{FD},\;\mathrm{GDP}\right)$$

The variables in the research were transformed into a natural logarithm. Following the pioneer study, Manning [56] recommended that normality problems be noticed in the variables before conversion to the logarithm form. The association between EF and other variables was inspected by employing the following model. Additionally, the study added GDP square (GDP^2^) to test the EKC hypothesis’s validity. The EKC hypothesis in its extended form is presented as follows.

(2) $${\mathrm{lnEF}}_{i,t}=\varphi_{0}+\varphi_{1}{\mathrm{lnNRR}}_{i,t}+\varphi_{2}{\mathrm{lnTI}}_{i,t}+\varphi_{3}{\mathrm{lnFD}}_{i,t}+\varphi_{4}{\mathrm{lnGDP}}_{i,t}+\varphi_{5}{\mathrm{lnGDP}}^{2}{}_{i,t}+\varepsilon_{i,t}$$

In Equation (2), EF indicates the total ecological footprint, NRR denotes natural resource rents, TI presents the technological innovation, FD demonstrates the financial development, GDP displays the economic growth per capita; and i and t signify the 90 BRI economies and given time dimension (1991–2018), respectively. Therefore, this study aims that NRR, and, aside from its direct influence, TI may play a moderating role on the EF in 90 BRI economies. Thus, this study explored the moderation effects between NRR and TI by focusing on this problem. Therefore, in Equation (3), we included a moderation effect term, which is written as follows:

(3) $${\;\mathrm{lnEF}}_{i,t}=\varphi_{0}+\varphi_{1}{\mathrm{lnNRR}}_{i,t}+\varphi_{2}{\mathrm{lnTI}}_{i,t}+\varphi_{3}{\mathrm{lnFD}}_{i,t}+\varphi_{4}{\mathrm{lnGDP}}_{i,t}+\varphi_{5}{\mathrm{lnGDP}}^{2}{}_{i,t}+\varphi_{6}{\mathrm{lnNR}*\mathrm{lnTE}}_{i,t}+\varepsilon_{i,t}$$

where $\varphi_{0}\;$ denotes the constant term; $\varepsilon_{i,t}\;$ displays the error term; and $\varphi_{1}$ → $\varphi_{6}$ represents the elasticity of candidate variables.

### 3.1. Data

This study explored the long-run association among NRR, TI, FD, and EF in 90 BRI economies using longitudinal data from 1991 to 2018. The name of the sample economies is provided in the Appendix A (see Table A1). The classification of these countries is based on the UN countries’ classification [57]. The variable EF is calculated in terms of global hectares per capita, a total of carbon, farmland, built-up land, forest land footprint, fishing grounds, and grazing land. NRR is the sum of forest rents, oil rents, coal rents, natural gas rentals, and mineral rents as a proportion of GDP. TI is determined by the number of patent applications. The current study used a relatively new FD measure established by the International Monetary Fund (IMF) [58]. This index assesses the accessibility, depth, and efficiency of financial markets and institutions. The economic growth is computed in terms of constant 2010 U.S. dollars. The GDP and NRR data were acquired from the World Development Indicator [59]. The data on EF were extracted from the Global Footprint Network [60], and the data on FD were sourced from IMF [58]. Finally, the TI data were collated from the World Intellectual Property Organization [61].

### 3.2. Methodologies Framework

This study adopted the following advanced econometrics methods: (i) We confirmed the cross-sectional dependence (CD) by employing the Pesaran CD method; (ii) augmented cross-sectional IPS (CIPS) panel unit root methods were employed to verify the stationary level of candidate variables; (iii) the study used the Westerlund cointegration method for the detection of log-run relationships among variables; and (iv) this research applied the AMG to examine the long-run elasticities of the variables.

#### 3.2.1. Cross-Sectional Dependence (CD) Test

The possible CD occurs due to externalities, geographical and financial globalization, spatial effects, economic integration, and individual-specific effects [5,62,63,64]. Therefore, it is important to investigate the CD issue, whether it occurs or not, among all cross-sections. Moreover, in a panel data study, examining CD is critical because failing to do so may result in ambiguous and biased outcomes. Therefore, to deal with the problem, we employed a CD test such as the Pesaran CD test proposed by Pesaran [65].

(4) $$\mathrm{CD}=\sqrt{\frac{2T}{N\left(N-1\right)}}\overset{N-1}{\underset{i=1}{\sum }}\overset{N}{\underset{j=i+1}{\sum }}\rho_{\mathrm{ij}}$$

where T signify time; N is the size of the panel data; and $\rho_{\mathrm{ij}}$ is the coefficient of correlation. The null hypothesis of the CD test is that there is no CD among the cross-sectional units. The alternative hypothesis is that CD exists among sample countries.

#### 3.2.2. Slope Homogeneity Test

After evaluating the CD, the next step is to look at the slope homogeneity between the cross-sections. The issue of heterogeneity is critical due to differences in the BRI countries’ demographic and economic structures. The consistency of panel estimators may be affected by variation in slope parameters. Because of this, this study employed the slope homogeneity approach proposed by Pesaran and Yamagata [66]. The test statistic’s equation is as follows:

(5) $${\tilde{\Delta }}_{\mathrm{SH}}={\left(N\right)}^{\frac{1}{2}}{\left(2K\right)}^{-\frac{1}{2}}\left(\frac{1}{N}\tilde{S}-k\right)$$

(6) $${\tilde{\Delta }}_{\mathrm{ASH}}={\left(N\right)}^{\frac{1}{2}}{\left(\frac{2k(T-k-1}{T+1}\right)}^{-\frac{1}{2}}\left(\frac{1}{N}\tilde{S}-k\right)$$

$\mathrm{where}\;{\tilde{\Delta }}_{\mathrm{SH}}$ represents the delta tilde and ${\tilde{\Delta }}_{\mathrm{ASH}}$ shows the corrected delta tilde.

#### 3.2.3. Panel Stationarity Test

The next phase in the econometric approach is to check the stationary/integration level of all involved variables after testing the CD of data. The first-generation unit root approaches such as Levin-Lin and Chu, I’m, Pesaran, and Shin (IPS) cannot solve the CD’s problem [64]. As a result, to account for the existence of CD, this research employed the second generation CIPS Pesaran [67]. The following are the CIPS test statistics:

(7) $${\Delta \mathrm{CA}}_{i,t}=\varphi_{i}+\varphi_{i}Z_{i,t-1}+\varphi_{i}{\bar{\mathrm{CA}}}_{t-1}+\overset{p}{\underset{l=0}{\sum }}\varphi_{\mathrm{il}}\Delta \bar{{\mathrm{CA}}_{t-1}}+\overset{p}{\underset{l=0}{\sum }}\varphi_{\mathrm{il}}{\Delta \mathrm{CA}}_{i,t-1}+\mu_{\mathrm{it}}$$

where ${\bar{\mathrm{CA}}}_{t-1}$ and $\Delta \bar{{\mathrm{CA}}_{t-1}\;}$ are the averages of the cross-sections. The statistics of the CIPS test are detailed in the study, as seen below:

(8) $$\hat{\mathrm{CIPS}}=\frac{1}{N}\overset{n}{\underset{i=1}{\sum }}{\mathrm{CDF}}_{i}$$

#### 3.2.4. Panel Cointegration Test

The next phase in the econometric process is to assess the long-run association between the variables. Westerlund [68] developed the second-generation cointegration test to find a long-run connection between the series. This test is superior compared to traditional cointegration approaches such as Kao and Pedroni as it gives unbiased estimates in the existence of CD and heterogeneity [69]. Westerlund tests comprise four types of test statistics such as Gt, Ga (group), and Pt, Pa (panel), which are estimated through Equation (9), described as follows:

(9) $$\alpha_{i}\left(L\right){\Delta y}_{\mathrm{it}}=\delta_{1i}+\delta_{2i}t+\alpha_{i}(y_{\mathrm{it}-1}-\beta_{i}^{\prime }x_{\mathrm{it}-1}+\lambda_{i}{\left(L\right)}^{\prime }v_{\mathrm{it}}+e_{\mathrm{it}}$$

where $\delta_{1i}=\alpha_{i}\left(1\right)\phi_{2i}-\alpha_{i}\phi_{1i}+\alpha_{i}\phi_{2i}$ and $\delta_{2i}=-\alpha_{i}\phi_{2i}$.

In Equation (9), $\beta_{i}$ is a coefficient of error correction and $\alpha_{i}$ is the direction of the cointegration relationship between x and y. The following are the test statistics:

(9a) $$G_{t}=\frac{1}{N}\overset{N}{\underset{i=1}{\sum }}\frac{\alpha_{i}^{\prime }}{\mathrm{SE}\left(\alpha_{i}^{\prime }\right)}$$

(9b) $$G_{a}=\frac{1}{N}\overset{N}{\underset{i=1}{\sum }}\frac{{T\alpha }_{i}^{\prime }}{\alpha_{i}^{\prime }\left(1\right)}$$

(9c) $$P_{t}=\frac{\alpha^{\prime }}{\mathrm{SE}\left(\alpha^{\prime }\right)}$$

(9d) $$\alpha^{\prime }=\frac{P_{a}}{T}$$

In Equation (9), the parameter for error correction $\left(\alpha^{\prime }\right)$ is calculated by putting the value of $P_{a}=\;T\alpha^{\prime }$. As a result, the error correction variable can be defined as $\left(\alpha^{\prime }\right)=\frac{P_{a}}{T}$. This shows that if there is a short-run disequilibrium, the proportion of error should be adjusted annually.

#### 3.2.5. Long-Run Estimation

Following this, we examined the long association between the NRR and EF in the existence of TI, FD, and economic growth. Economists suggest a number of methodologies for analyzing panel data. However, previous research used first-generation cointegration approaches (FMOLS, DOLS, ARDL, and so on), which might lead to biased outcomes in the existence of CD and heterogeneity. Thus, the AMG technique was used in the study by Eberhardt [70]. This method is worthy for a variety of reasons. This technique is appropriate in the case of endogeneity, non-stationary, CD, and heterogeneity. Furthermore, this method takes into account the correlation, particularly among cross-sections. The AMG equation is as follows:

(10) $${\Delta \mathrm{EF}}_{\mathrm{it}}=\varphi_{0}+\varphi_{1}{\Delta \mathrm{NRR}}_{\mathrm{it}}+\varphi_{2}{\Delta \mathrm{TI}}_{\mathrm{it}}+\varphi_{3}{\Delta \mathrm{FD}}_{\mathrm{it}}+\varphi_{4}{\Delta \mathrm{GDP}}_{\mathrm{it}}+\overset{T}{\underset{t=2}{\sum }}p_{t}\left({\mathrm{AD}}_{t}\right)+\mu_{\mathrm{it}}$$

where ${\mathrm{AD}}_{t}$ indicates the first difference; T−1 dummies for the time; and j  specifies dummy time parameters. The next step is where $p_{t}$ is substituted with the  τ variable, demonstrating the standard dynamic process as:

(11) $${\Delta \mathrm{EF}}_{\mathrm{it}}=\varphi_{0}+\varphi_{1}{\Delta \mathrm{NRR}}_{\mathrm{it}}+\varphi_{2}{\Delta \mathrm{TI}}_{\mathrm{it}}+\varphi_{3}{\Delta \mathrm{FD}}_{\mathrm{it}}+\varphi_{4}{\Delta \mathrm{GDP}}_{\mathrm{it}}+d_{1}\left(\lambda_{t}\right)+\mu_{\mathrm{it}}$$

(12) $${\Delta \mathrm{EF}}_{\mathrm{it}}-\lambda_{t}=\varphi_{0}+\varphi_{1}{\Delta \mathrm{NRR}}_{\mathrm{it}}+\varphi_{2}{\Delta \mathrm{TI}}_{\mathrm{it}}+\varphi_{3}{\Delta \mathrm{FD}}_{\mathrm{it}}+\varphi_{4}{\Delta \mathrm{GDP}}_{\mathrm{it}}+\mu_{\mathrm{it}}$$

First, the group-specific regression model was reformed with $\varphi_{t}$ and following that, the averages of the group-specific models were calculated. This research applied the common correlated effect mean group (CCEMG) approach to test the robustness of the Pesaran model [71].

## 4. Results and Discussion

The first econometric step of empirical analysis scrutinizes the existence of CD among the variables. The results of the CD tests of the null hypothesis (H_0_) of no CD between the variables are given in Table 1. According to the CD test proposed by Pesaran [65], it is rejected at the 1% significance level. This specifies that an erratic shock (positive/negative) in one country will affect the other countries in the BRI region. The CD was further corroborated by absolute mean values ranging from 0.445 to 0.829. In contrast, BRI economies showed a heterogenous slope due to the different growth patterns. As shown in Table 2, the BRI economies’ panel has various degrees of technological advancement and development. As a result, the slope homogeneity test findings indicate that the model has a data heterogeneity problem.

After testing the CD, there is a important requirement to find the integration order/stationary level of the variables. To do this, we applied CIPS unit root tests. Table 3 shows the CIPS unit root test outcomes, which revealed that EF, NRR, and FD were not stationary at these levels, showing that they cannot reject the null hypothesis. However, they became stationary at their first difference at a 1% significance level. These findings reveal that all the candidate variables are stationary, and it is appropriate to assess the long-run cointegration of the variables.

In order to verify the long-run equilibrium between the variables, we used the second-generation test, namely, the Westerlund [68]. Table 4 explores the Westerlund cointegration test outcomes. The findings of the Westerlund tests show that all four models had a long-run cointegration association. The outcomes in the high- and middle-income BRI region demonstrate robust likelihood values; they failed to reject the null hypothesis (H_0_) of no cointegration for G_a_. In contrast, the outcomes for G_t_, P_t_, and P_a_ provide an appropriate indication to reject the H_0_ with corresponding probability levels that are significant. Therefore, it shows that all variables comprise a long-run cointegration.

We can assess the long-run relationship via the AMG method after completing the cointegration examination between the variables. The results of the AMG test are demonstrated in Table 5. The NRR and the EF had a positive and significant association in Model 1 for the BRI full panel. Accordingly, a 1% increase in NRR in BRI economies led to driving an EF of about 0.348%. The average share of NRR in BRI economies has been increased approximately 1.968% from the years between 1991 to 2018 based on the rents for oil, natural gas, coal (hard and soft), mineral rentals, and forest rents [72]. These BRI economies are putting stress on NRR reserves to achieve energy demand, enhancing the pressure on the environment. Considering these facts, we conclude that investment in clean energy (eco-friendly technology) should be an essential element in reducing the EF. The elasticity of TI was also significant and had a negative influence on EF, demonstrating that a 1% change in TI was associated with a 0.088 percent decrease in EF. Thus, the TE is a significant element for sustainable development, attaining energy efficiency and supporting a low EF. These results align with those in [5,73].

The coefficient of FD has a positive impact on the EF, so the 0.043% change in the EF is due to FD. Therefore, the policymakers should encourage foreign direct investment (FDI) to encourage investors to develop eco-friendly technologies and pollution-free industries. These findings coincide with those in [74]. Furthermore, in Model 1 of BRI countries, GDP and GDP^2^ positive and negative values with EF indicate the EKC hypothesis’s validity with an inverted U-shape. Specifically, a 1% upsurge in GDP enhances the EF by 0.776%, while a 1% rise in GDP^2^ lessens the EF by 0.028%. These findings align with [9,20]. According to Model 2 of the BRI full panel, the negative coefficient of the interaction term (NRR* TI) shows that TI negatively moderates the association between NRR in reducing EF, which means when NRR improves the environmental quality due to the promotion of TI. 

Following Model 1 of high income BRI economies, the coefficient of NRR has a significant positive impact on EF at a 1% significance level, indicating that a 1% increase in NRR results in a 0.422 percent rise in EF. Since 2000, overall energy demand has grown by more than 83%, and the share of this development has been happening by a doubling consumption of fossil fuel utilization in BRI economies. Oil is also the primary source of power generation/energy demand in this region, leading to worse quality of the environment. This subset of results was discovered similarly in [4,6,13]. Alternatively, in the case of high income, the coefficient value of TI had a negative and statistically significant impact in BRI economies; particularly, a 1% rise in TI resulted in a 0.068% drop in EF. Specifically, TI comprises the development of new ideas, adjustment/modification of the current production process, and an essential solution for sustainable development and environmental issues [75]. Our results are consistent with the findings of [76].

Additionally, a 1% rise in FD will result in a 0.037 percent decrease in the quality of the environment in the long-run. This empirical evidence indicates that FD enhanced the EF because most BRI countries rely on fossil fuels. These findings are in accordance with [20,77,78]. Conversely, GDP and GDP^2^ elasticity were 0.837 and −0.025, respectively; this supports the EKC hypothesis with a U-shape. It was found that a 1% rise in GDP corresponded to a 0.837 percent surge in EF, while a 1% rise in GDP^2^ decreased EF by −0.025%. This means that pollution level increases during the early stages of economic growth. However, after arriving at a specific point, the pollution level will decrease. This result is congruent with the findings of [5,79]. Moreover, according to Model 2 of the high income BRI economies, the negative coefficient of an interactive term (NRR* TI) shows that TI negatively moderates the relationship between NRR and EF. 

It was noted that a 1% upsurge in NRR* TI led to a 0.033% reduction in EF. In this regard, the result of NRR in middle income BRI economies had a significant and positive effect on the EF in Model 1. Specifically, at the 1% significance level, a 1% rise in NRR enhanced the EF by 0.222 percent in the long-run. This finding backs up the hypothesis that NRR is the primary source of environmental damage. The outcome supports the hypothesis that NRR is the primary source of environmental damage in the region. The positive impact of NRR can be supported by the fact that middle-income countries have the greatest number of oil-producing economies. These countries are placing immense pressure on their NRR to meet their energy needs, causing environmental damage. The findings of the present empirical evidence are consistent with those of [5].

Furthermore, the TI coefficient has a negative and significant effect on EF, revealing that a 1% rise in TI reduced the EF by −0.055%. Thus, the TI outcomes minimized the EF through eco-friendly technologies and supported the existing findings Wang et al. [12,80], in contrast with [5]. The elasticity of FD was also significant and negative, indicating that a 1% influence in FD reduced the EF by 0.031%. This outcome is similar to those found by [20,81,82]. In order to support this result, Ahmad et al. [79] suggested that FD creates environmentally friendly technologies and current inventive production practices, which boost economic growth while lowering EF.

Likewise, the validity of the EKC hypothesis is expressed by the positive and negative values of GDP and GDP^2^ on EF in middle income BRI countries. The findings are consistent with those of [5,83]. Moreover, Model 2 of middle income BRI countries shows the significant moderation effect of NRR with TI on EF. The outcome of the interaction term (NRR* TI) revealed that it had a significant and adverse effect on the EF. Notably, a 1% rise in NRR* TI resulted in a −0.029 percent decrease in EF. Ahmad et al. [5] contended that economic activities and trade openness enhance energy utilization, and revolutions in technology improve the efficient utilization of NRR and energy efficiency; thus, it helps mitigate EF.

Considering Model 1 of low income BRI economies, the NRR coefficient affected EF positively and significantly. It is worth noting that a 1 percent increase in NRR outcomes led to a 0.188 percent rise in EF. The low income BRI economies hold almost 20% of the world’s proven oil reserves and put massive pressure on their NRR assets to accomplish their energy demand, decreasing environmental quality [6]. These results are also in line with [5]. The concept of the inverted U-shaped EKC hypothesis is expressed by the positive and negative values of the GDP and GDP^2^ coefficients with EF. It is worth noting that a 1% increase in GDP results in a 0.520% rise in EF, whereas a 1% upsurge in GDP^2^ results in a 0.020% decline in EF. These results are comparable with [84,85]. Accordingly, in Model 2 for low income BRI countries, the result of the interactive term (NRR* TI) had a significantly negative impact on EF, demonstrating that TI could improve the utilization of NRR and enhance the environmental quality through eco-friendly technologies. More specifically, a 1% influence in NRR* TI will decrease the EF by 0.015%. However, we observed that NRR (without interaction with TI) was significant in all models except Model 2 of middle income BRI countries, with no significant association with EF in contrast (with interaction term). It appears to have a significant influence on EF. 

### Robustness Check

The robustness of the results above-mentioned was tested using the alternative measure of EF with GHG as a dependent variable and an alternative estimator (i.e., CCEMG) [71]. According to Table 6, the results of NRR significantly lower the quality of the environment while TI enhances environmental sustainability. In addition, in the case of a full panel of BRI countries, the findings likewise corroborate the inverted U-shaped EKC hypothesis. Furthermore, the interaction term between NRR and TI (NRR × TI) has a statistically negative influence on GHG emissions, similar to the findings of the AMG estimators. Hence, our results are robust and reliable to both robustness checks (alternative variable and method), which ensures the accuracy of our findings.

## 5. Conclusions

For the past couple of decades, a sustainable environment has been a desired state worldwide. Environmental pollution can occur as a result of a variety of economic activities. Various socio-economic issues have a positive or negative impact on the environment. Many earlier studies have resulted in efficient input allocation as an environmentally beneficial component. This research explored the impact of NRR, TI, and FD with the interaction term (NRR* TI) on the EF from 1991–2018 in BRI countries in the EKC hypothesis framework. Furthermore, to capture the effect of income differences, this study divided the samples of the BRI countries into three income groups. The EF, a complete proxy (based on six different environmental indicators), quantifies environmental quality in the current study. The Pesaran CD technique was used to check the CD. The stationary level was investigated using the CIPS panel unit root technique. The cointegration method, developed by Westerlund [68], determines whether there is a long-run link among the variables under consideration. The AMG estimator was employed in this research to estimate the long-run elasticity of variables. The findings show that a NRR has a positive effect on EF, while TI and interaction with NRR (NRR*TI) negatively impact the EF in the BRI countries. Interestingly, the same outcomes were found in three income groups regarding the association between NRR, TI, and interaction terms, but at a different magnitude and significance level. These findings reveal that NRR reserves are needed in order to meet the energy demand, putting further strain on the environment. Given these facts, we infer that investing in clean energy (environmentally technologies) should be a key component in lowering the EF. Furthermore, results confirm the presence of the income-wise EKC hypothesis for BRI countries. 

### Policy Implication for Environmental Sustainability

According to the findings, this research recommends the following policy implications for stakeholders, governments, and policymakers in general, and specifically to BRI economies for environmental sustainability. Policymakers in BRI countries should move resources away from resource-rich sectors of industries/manufacturing sectors to enhance/promote economic growth and use these NRRs efficiently for a progressive, sustainable environment. TI should be massively utilized to use energy and NRR efficiently.

The FD process should not be ignored in the policy framework for a sustainable environment in BRI economies. A balance should be struck between the economic benefits of FD and environmental quality. Furthermore, the policymakers should encourage clean and green foreign investment and welcome these investments, which carry technical skills, environmentally friendly technologies, and carbon-free methods in the BRI economies. 

These countries should change their dirty energy strategies into renewable energy sources. The government of these countries should focus on trade promotion with advanced technology by supplying eco-friendly technology and sources of renewable energy including hydropower, solar, wind, biomass, geothermal heat, waste-based energy, and employ environmental regulation policies such as imposing carbon tax/quota system on emission-intensive products, which could help to maximize environmental quality. Environmentally friendly technologies will preserve the BRI countries’ international capacities while ensuring a long-term environmentally sustainable economy.

The enormous increase in EF and the economic performance of various economies continue to pique the interest of academics and practitioners. Concerns about global warming and its impact on human and animal health, hence on sustainable development, are also growing. As a result, policymakers and academics must examine the critical significance of absorptive capacity in promoting sustainable development.

This research has some shortcomings that should be addressed in future research. Because our analytical approach does not take into account crucial cultural and social aspects, future researchers could expand on this research by investigating the interaction role of institutional quality and NRR in the pollution haven or halo hypothesis framework, making a significant contribution to the literature and will ultimately help to curb environmental pollution.

## Figures and Tables

**Table 1:** Cross-sectional dependence test results of BRI economies.

Variables	Statistic	*p*-Value	Abs (corr)
EF	13.468 ***	0.000	0.483
NRR	25.176 ***	0.000	0.685
TI	29.853 ***	0.000	0.445
FD	21.280 ***	0.000	0.657
GDP	65.535 ***	0.000	0.819
GDP^2^	65.920 ***	0.000	0.829

Note: *p* < 0.01, indicate *** espectively.

**Table 2:** Results of slope homogeneity test in BRI economies.

Test	BRI (Full Panel)		High-Income		Middle-Income		Low-Income	
	Value	*p*-Value	Value	*p*-Value	Value	*p*-Value	Value	*p*-Value
$\tilde{\Delta }$	16.890 ***	0.000	17.809 ***	0.000	14.593 ***	0.000	13.373 ***	0.000
${\tilde{\Delta }}_{adjusted}$	17.671 ***	0.000	18.538 ***	0.000	15.257 ***	0.000	16.417 ***	0.000

Note: *p* < 0.01, indicate ***, respectively.

**Table 3:** CIPS panel unit root test result in BRI economies.

Variable	I(0)		I(1)		Order
	Intercept	Intercept & Trend	Intercept	Intercept & Trend	
EF	−1.824	−1.872	−3.519 ***	−3.935 ***	I(1)
NRR	−1.989	−1.828	−3.673 ***	−3.786 ***	I(1)
TI	−2.909 ***	−3.645 ***	-	-	I(0)
FD	−2.497	−1.489	−4.654 ***	−4.237 ***	I(1)
GDP	−2.385 **	−2.762 **	-	-	I(0)
GDP^2^	−2.193	−2.760 **	-	-	I(0)

Note: *p* < 0.01, 0.05, and indicate ***, **, and respectively.

**Table 4:** Westerlund panel cointegration test results in BRI economies.

	G_t_	G_a_	P_t_	P_a_
BRI (full panel)	−3.165 ***	−12.965 **	−17.676 ***	−14.895 ***
	[−9.517]	[−1.946]	[−8.998]	[−5.974]
High-income	−3.218 ***	−11.755	−16.321 ***	−13.859 ***
	[−9.844]	[−1.479]	[−6.839]	[−4.772]
Middle-income	−3.565 ***	−11.357	−14.291 ***	−13.496 ***
	[−8.824]	[−0.740]	[−5.994]	[−4.478]
Low-income	−4.453 ***	−13.272 **	−20.955 ***	−14.876 ***
	[−10.857]	[−2.796]	[−10.975]	[−5.953]

Note: *p* < 0.01, 0.05, and indicate ***, **, and respectively. [] is for Z-value.

**Table 5:** Results of augmented mean group test results.

Variables	BRI (Full Panel) (1)	BRI (Full Panel) (2)	High-Income (1)	High-Income (2)	Middle-Income (1)	Middle-Income (2)	Low-Income (1)	Low-Income (2)
NRR	0.348 ***	0.488 ***	0.422 ***	0.480 ***	0.222 ***	0.2752	0.188 ***	0.314 ***
	[0.134]	[0.126]	[0.142]	[0.060]	[0.068]	[0.2758]	[0.071]	[0.044]
TI	−0.088 **	−0.084 **	−0.068 *	−0.068 **	−0.055 **	−0.042 **	−0.044 *	−0.021 ***
	[0.036]	[0.035]	[0.039]	[0.032]	[0.021]	[0.018]	[0.024]	[0.007]
FD	0.043 ***	0.070 **	0.037 **	0.044 **	−0.031 **	−0.043 **	0.034 **	0.011 ***
	[0.019]	[0.027]	[0.019]	[0.020]	[0.011]	[0.016]	[0.014]	[0.003]
NRR*TI	---	−0.039 *	---	−0.033 ***	---	−0.029*	---	−0.015 ***
		[0.024]		[0.007]		[0.015]		[0.005]
GDP	0.776 ***	0.852 ***	0.837 ***	0.786 **	0.497 ***	0.449 ***	0.520 ***	0.480 ***
	[0.134]	[0.148]	[0.125]	[0.365]	[0.082]	[0.077]	[0.099]	[0.060]
GDP^2^	−0.028 ***	---	−0.025 ***	---	−0.018 ***	---	−0.020 ***	---
	[0.006]		[0.008]		[0.005]		[0.007]	
Constant	−2.184 **	−3.029 ***	−2.558 ***	−2.525 **	−2.353 **	−2.831 ***	−2.447	−2.227 **
	[0.848]	[1.007]	[0.978]	[1.172]	[1.108]	[1.057]	[1.359]	[1.133]

Note: *p* < 0.01, 0.05, and 0.10 indicate ***, **, and *, respectively. [] is for standard error.

**Table 6:** Robustness checks with the CCEMG method (full sample).

	Model 1			Model 2		
Variables	GHG	Std. Err.	*p*-Value	GHG	Std. Err.	*p*-Value
NRR	0.622 **	0.272	0.019	0.592 **	0.241	0.014
TI	−0.066 ***	0.010	0.000	−0.022 ***	0.008	0.000
FD	0.055 ***	0.015	0.010	0.041 **	0.017	0.025
NRR*TI	--------			−0.081 ***	0.020	0.001
GDP	0.744 *	0.391	0.061	0.762 **	0.310	0.014
GDP^2^	−0.081 ***	0.031	0.009	---------		
Constant	−2.599 ***	0.698	0.000	−2.695 ***	0.589	0.000

Note: *p* < 0.01, 0.05, 0.10 indicate ***, ** and *, respectively.

## Data Availability

The datasets used and/or analyzed during the current study are available from the corresponding author on reasonable request.

## Appendix A

**Table A1:** List of Belt and Road Initiative countries.

Sr #	Name of Country	Income Level	Sr #	Name of Country	Income Level
1	Albania	MI	46	Luxembourg	HI
2	Algeria	MI	47	North Macedonia	MI
3	Angola	LI	48	Malaysia	MI
4	Armenia	MI	49	Maldives	MI
5	Austria	HI	50	Malta	HI
6	Azerbaijan	MI	51	Moldova	LI
7	Bahrain	HI	52	Mongolia	LI
8	Bangladesh	LI	53	Morocco	LI
9	Belarus	MI	54	Mozambique	LI
10	Benin	LI	55	Namibia	MI
11	Bolivia	LI	56	Niger	LI
12	Bulgaria	MI	57	Nigeria	LI
13	Cambodia	LI	58	Oman	HI
14	Cameroon	LI	59	Pakistan	LI
15	Chile	HI	60	Panama	HI
16	Congo, Dem. Rep.	LI	61	Peru	MI
17	Costa Rica	MI	62	Philippines	LI
18	Croatia	HI	63	Poland	HI
19	Czech Republic	HI	64	Portugal	HI
20	Dominican Republic	MI	65	Qatar	HI
21	Ecuador	MI	66	Korea, Rep.	HI
22	Egypt, Arab Rep.	LI	67	Romania	MI
23	El Salvador	LI	68	Russian Federation	MI
24	Estonia	LI	69	Saudi Arabia	HI
25	Ethiopia	LI	70	Senegal	LI
26	Gabon	MI	71	Serbia	MI
27	Georgia	MI	72	Singapore	HI
28	Ghana	LI	73	Slovak Republic	HI
29	Greece	HI	74	Slovenia	HI
30	Grenada	MI	75	South Africa	MI
31	Guyana	MI	76	Sri Lanka	MI
32	Hungary	HI	77	Suriname	MI
33	Indonesia	LI	78	Tanzania	LI
34	Iran, Islamic Rep.	MI	79	Thailand	MI
35	Italy	HI	80	Togo	LI
36	Jamaica	MI	81	Tunisia	LI
37	Jordan	MI	82	Turkey	MI
38	Kazakhstan	MI	83	Turkmenistan	MI
39	Kenya	LI	84	Ukraine	LI
40	Kuwait	HI	85	U.A.E	HI
41	Kyrgyz Republic	LI	86	Uruguay	HI
42	Latvia	HI	87	Uzbekistan	LI
43	Lebanon	MI	88	Venezuela, RB	MI
44	Libya	MI	89	Vietnam	LI
45	Lithuania	HI	90	Zambia	LI
